# Genipin as an Effective Crosslinker for High-Performance and Flexible Direct-Printed Bioelectrodes

**DOI:** 10.3390/molecules31020327

**Published:** 2026-01-17

**Authors:** Kornelia Bobrowska, Marcin Urbanowicz, Agnieszka Paziewska-Nowak, Marek Dawgul, Kamila Sadowska

**Affiliations:** Nalecz Institute of Biocybernetics and Biomedical Engineering, Polish Academy of Sciences, Ks. Trojdena 4 St., 02-109 Warsaw, Poland; murbanowicz@ibib.waw.pl (M.U.); apaziewska@ibib.waw.pl (A.P.-N.); mdawgul@ibib.waw.pl (M.D.); ksadowska@ibib.waw.pl (K.S.)

**Keywords:** FAD-dependent glucose dehydrogenase, mediated electron transfer, genipin, direct printing

## Abstract

The development of efficient bioelectrodes requires suitable fabrication strategies, starting with the electrode material, which affects the electron transfer between the biocatalyst and the electrode surface. Then, selection and adjustment of the enzyme immobilization conditions are essential to enhance the performance of the bioelectrodes for their desirable utility. In this study, we report the fabrication of a high-performance bioelectrode using a one-step crosslinking of FAD-dependent glucose dehydrogenase (FAD-GDH) and thionine acetate as a redox mediator, with genipin serving as a natural, biocompatible crosslinker. Electrodes were manufactured on flexible polyester substrates using a direct printing technique, enabling reproducible and low-cost production. Among the tested crosslinkers, genipin significantly enhanced the catalytic performance of bioelectrodes. Comparative studies on graphite, silver, and gold electrode materials identified graphite as the most suitable due to its extended electroactive surface area. The developed bioelectrodes applied to glucose biosensing demonstrated a linear amperometric response to glucose in the range of 0.02–2 mM and 0.048–30 mM, covering clinically relevant concentrations. The application of artificial sweat confirmed high detection accuracy. These findings highlight the potential integration of genipin-based enzyme–mediator networks for future non-invasive sweat glucose monitoring platforms.

## 1. Introduction

In recent years, flexible printed electrodes have attracted much attention due to their lightweight and beneficial mechanical properties, such as high extensibility [1,2]. They have a wide range of potential applications, such as energy storage, sensors, and healthcare devices [3], while their flexibility opens up the possibility of applying them in wearable devices. There are a number of technologies for electrode material deposition, such as screen printing, direct printing, and ink jetting. Direct printing is a low-cost and high-resolution method that enables rapid prototyping of electrochemical devices; nevertheless, optimization of process parameters is crucial to ensure the reproducibility and reliability of printed electrode systems [4,5]. The adjustability and tunability of direct printing technology enable the utilization of various types of substrate materials to achieve tailored mechanical features in the final electrode system.

The integration of redox enzymes with the electrodes offers a vast opportunity to design new bioelectronic systems for medical diagnostics, environmental monitoring, and bioelectricity generation. The efficient direct or mediated electron transfer between the enzyme active site and the electrode surface is a key determinant of performance. Effective electrocatalysis requires the seamless integration of the electrode substrate with a well-structured biolayer, wherein critical factors such as the enzyme immobilization method and the choice of electron transfer pathway—direct or mediated—determine the overall bioelectrode outcome [6]. One of the effective ways to improve the performance and stability of bioelectrodes is the selection of an appropriate enzyme immobilization method [7]. So far, various protocols, including adsorption, covalent binding [8], crosslinking, and entrapment/encapsulation, have been applied [9]. In crosslinking, molecules are covalently interconnected by the linker agent, which forms spacers between two adjacent biomolecules [10]. The orientation of crosslinked enzymes, especially spatial orientation and inter-enzyme distance, is a key factor in enhancing the reaction rate and increasing the total catalytic efficiency of enzymes [11].

Genipin is a naturally occurring, biocompatible crosslinker isolated from the fruit of the *Gardenia jasminoides* [12]. It is approximately 5000–10,000 times less cytotoxic than glutaraldehyde [13]. In the presence of oxygen, genipin reacts with two primary amine groups. The crosslinking mechanism under neutral aqueous conditions has been extensively discussed in the literature. In the proposed reaction pathway, the dihydropyran ring of genipin undergoes nucleophilic attack by a primary amine, leading to the formation of a tetrahydropyridine intermediate. Subsequently, a second, slower nucleophilic attack by another amine occurs at the carbonyl group of the methyl ester, resulting in the formation of a stable amide bond [14,15]. It is a commonly used crosslinking agent for biomaterials to improve their stability. The low toxicity of genipin makes it an ideal choice for preparing biocompatible enzyme networks [16]. Genipin was mainly used for crosslinking enzymes with additional support, like gelatin or chitosan, to form a hydrogel or to entrap the enzyme [17,18,19,20,21,22]. Nonetheless, biolayers obtained directly by genipin-based enzyme crosslinking have been rarely reported [16]. Cui et al. described glucose oxidase–catalase crosslinking using genipin, which resulted in an improved biocatalytic efficiency by reducing the distance between enzymes [11]. Glucose oxidase was immobilized using genipin on plasma-treated fibrous carbon felt and evaluated for wastewater treatment in the work of Kahoush et al. [14]. After crosslinking, improved enzyme stability was reported.

Glucose oxidase (GOx) and glucose dehydrogenase (GDH) are the most common enzymes for the anodic glucose oxidation in enzymatic biofuel cells (EBFCs) and for glucose determination in enzymatic biosensors [23,24]. GOx uses oxygen as an electron acceptor and generates hydrogen peroxide during glucose oxidation, which has an inhibitory effect on the enzyme’s catalytic activity [25]. Therefore, insensitive to oxygen, glucose dehydrogenase can be used as an alternative enzyme. GDH can be categorized based on the type of coenzyme. In comparison to nicotinamide adenine dinucleotide (NAD) and pyrrolo-quinoline quinone (PQQ), flavin adenine dinucleotide (FAD)-dependent GDH (FAD-GDH) demonstrates high glucose specificity, as well as thermal and pH stability [26,27]. Considering the properties of FAD-GDH and the availability of various redox mediators, FAD-dependent glucose dehydrogenase is currently a strongly explored enzyme in biocatalysis [28].

Selection of the appropriate redox mediator is pivotal for the successful construction of bioelectrodes. Previously reported redox mediators include osmium [29] and ruthenium complexes [30], quinone [31,32], and phenothiazine derivatives [33]. Cohen et al. tested the bioelectrocatalytic activity of FAD-GDH from *Talaromyces Emersonii* under a variety of redox mediators, including dichlorophenolindophenol, thionine acetate, and 2,3-dichloro-naphthoquinone (DCNQ), and in direct electron transfer (DET) configuration. The bioelectrodes were obtained on a glassy carbon electrode (GCE) modified with multiwalled carbon nanotubes (MWCNTs). Mediated electron transfer (MET) configuration yielded higher currents compared to DET, reaching the highest current density with DCNQ as a mediator. The developed bioelectrodes showed a linear response to glucose concentration in the 0–20 mM range [27]. Tsujimura and co-workers reported the electrochemical reductive grafting of Azure A onto the MWCNTs electrode. The three-dimensional porous structure of the MWCNTs electrode was determined to be favorable for enzyme immobilization, and efficient electron transfer was provided via the grafted Azure-A. The current density increased proportionally with an increase in the MWCNTs’ loading, reaching 5.6 mA·cm^−2^ for 200 μg of MWCNTs. However, bioelectrocatalytic current was measured at quite a high potential equal to 0.3 V vs. Ag/AgCl. The glucose oxidation activity on the bioelectrodes decreased with storage time, providing around 30% of the initial activity after 10 days of storage [34]. Tahar et al. designed a bioelectrode based on glucose dehydrogenase, NADP^+^, and MWCNTs coated with poly(methylene green) (MWCNTs-PMG). The electrochemical study shows that the bioanode exhibits fast electron transfer with performance against a low potential (0 V) during glucose oxidation. The long-term stability of bioelectrodes was attributed to a three-dimensional structure of MWCNTs-PMG [35]. Nevertheless, the above-presented bioelectrodes required more complex synthesis procedures compared to the crosslinking, which is a simple and efficient way of enzyme immobilization with a mediator. Hossain et al. crosslinked FAD-GDH via poly(ethylene glycol) diglycidyl ether (PEDGE) with four different phenothiazine mediators, including azure A, toluidine blue, methylene blue, and thionine, and examined their catalytic effect on bioelectrode performance. The highest catalytic current density during glucose oxidation (0.400 mA·cm^−2^ in 200 mM glucose) was generated on a bioelectrode prepared with thionine, since this mediator has two primary amine groups that were covalently crosslinked with free amine groups (on lysine) of FAD-GDH [33]. This research author also verified the impact of different crosslinkers and crosslinker spacer arm length, including epoxy, *N*-hydroxysuccinimide (NHS), and aldehydes, on bioelectrode performance. An adequate crosslinker spacer arm length affects the faster electron transfer, resulting in producing a catalytic current density of 0.830 mA·cm^−2^ in 200 mM glucose by FAD-GDH and a thionine network based on the NHS crosslinker [36] (see Table S1).

**Scheme 1:**
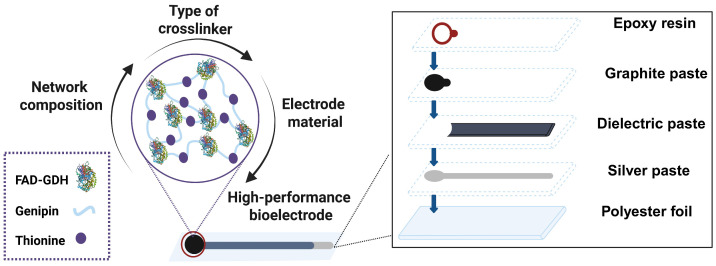
Schematic illustration of bioelectrode optimization (**left**) and manufacturing of graphite direct-printed electrode (**right**). Created with BioRender.com.

Herein, we integrated direct-printed electrodes with a FAD-dependent glucose dehydrogenase, crosslinked by genipin with thionine as a mediator. Catalytic current density generated during oxidation in a 200 mM glucose solution was equal to 0.942 mA·cm^−2^. Crosslinking with genipin enables more efficient electron transfer in the enzymatic network, resulting in 2.5 and 25 times higher current density compared to bioelectrodes obtained with bis(sulfosuccinimidyl) suberate (BS3) and glutaraldehyde (GA), respectively. Bioelectrodes were developed on direct-printed electrodes on a flexible substrate (Scheme 1), showing high reproducibility (relative standard deviation, *RSD*, 2.69%, calculated for 10 bioelectrodes) and were effectively applied to glucose biosensing, exhibiting a linear response in the concentration range between 0.048 and 30 mM at pH 8.

## 2. Results and Discussion

### 2.1. Optimization of Bioelectrode Performance

The electrochemical properties of free thionine acetate and thionine crosslinked in the FAD-GDH-genipin-thionine (FAD-GDH-G-TH) network were investigated using a graphite direct-printed working electrode. The solutions of TH were prepared in 100 mM acetate buffer (AC) at pH 5 and in 100 mM phosphate buffer (PB) at pH 6, 7, and 8. In the same buffer solutions, the electrochemical characteristics of the thionine crosslinked in the FAD-GDH-G-TH network were examined. The midpoint potential of free thionine has changed linearly by about 35 mV·pH^−1^ vs. Ag/AgCl|sat.KCl, moving to a more negative potential with increasing pH. Similar to free thionine, the midpoint potential of the crosslinked mediator has changed by ca. 34 mV·pH^−1^ vs. Ag/AgCl|sat.KCl, with a slight decrease to a more negative potential compared to the midpoint potential of the free thionine (Figure S1). According to the Nernst equation, a change of about 30 mV·pH^−1^ confirms that the redox process involves the transfer of two electrons. The acetate ion and one electron participate in the initial step of the redox reaction, followed by the involvement of one electron and one proton [37].

**Figure 1:**
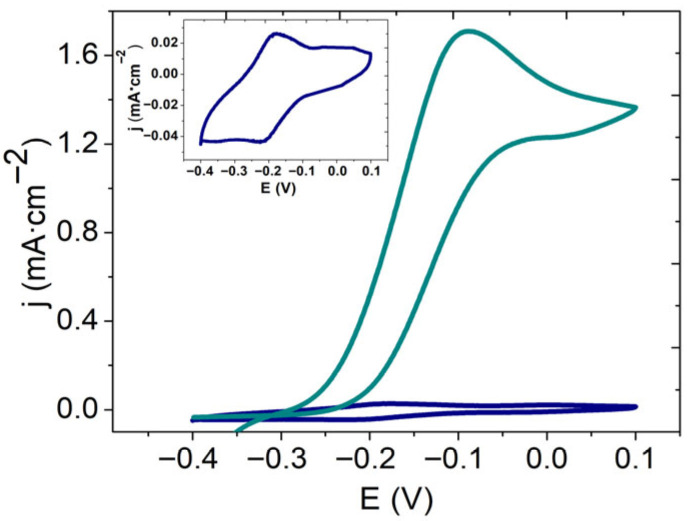
Cyclic voltammograms of the FAD-GDH-G-TH bioelectrode in the absence (navy; inset provides a magnified view) and in the presence (dark cyan) of 200 mM glucose in 100 mM PB, pH 8. Scan rate 5 mV·s^−1^.

The cyclic voltammogram of the FAD-GDH-G-TH bioelectrode recorded in the absence of glucose (Figure 1, navy curve) shows only the redox peaks of thionine between −0.178 and −0.212 mV vs. Ag/AgCl|sat.KCl. In the presence of glucose, the cyclic voltammogram (Figure 1, dark cyan curve) shows a remarkable change to a sigmoidal shape and a high increase in current density. The observed change in the shape and current provides the robust confirmation of the biocatalytic effect of glucose oxidation on the bioelectrode and successful incorporation of FAD-GDH into the crosslinked matrix. The schematic for the electron transfer pathway, illustrating the sequence during glucose oxidation on the electrode surface, is presented in Scheme S1.

Since the electrochemical properties of thionine, stability, activity of FAD-GDH, and crosslinking degree by genipin are pH-dependent [38], the influence of the pH on crosslinking efficiency and bioelectrode performance was verified. Mixtures of thionine, genipin, and FAD-GDH were prepared in 100 mM AC at pH 5 and in 100 mM PB at pH 6, 7, and 8. These mixtures were then drop-casted onto graphite direct-printed electrodes. Figure S2A presents the dependency between the currents generated during glucose oxidation on bioelectrodes crosslinked at different pHs. The highest current density was generated by the bioelectrode prepared in 100 mM PB at pH 7, indicating this pH as the most suitable for effective crosslinking and maintaining enzyme activity. To separate the effect of immobilization conditions from the operating conditions, firstly, the crosslinking pH of the FAD-GDH-G-TH bioelectrode was optimized, and then the pH of the glucose solution was optimized. Bioelectrodes crosslinked in PB at pH 7 delivered the highest steady-state currents, whereas crosslinking at pH 8 gave lower currents, consistent with reduced retained FAD-GDH activity after prolonged immobilization near the upper edge of its stability window (stable pH 5–7.5 at 25 °C). With the crosslinking pH fixed at 7, the operating pH (5–8) in acetate and phosphate buffers was evaluated (Figure S2B). The maximum catalytic response was obtained in glucose solution in 100 mM PB at pH 8, which agrees with the enzyme’s activity profile around neutral/slightly basic pH and with the pH-dependent shift in thionine’s midpoint potential. Therefore, subsequent measurements were performed in glucose solution in 100 mM PB at pH 8, while all bioelectrodes were prepared by crosslinking at pH 7. The impact of the temperature on the crosslinking process was also verified. Two series of bioelectrodes (*n* ≥ 3) were prepared in the same manner. One series of electrodes was stored for 16 h at 25 °C, and another at 4 °C. Bioelectrodes maintained at a lower temperature during the crosslinking process generated a 42.5% higher current density during glucose oxidation. It was reported that temperature has an impact on the crosslinking degree by genipin, with lower temperature reducing the crosslinking process [39]. On the other hand, slower enzyme and mediator crosslinking can ensure appropriate enzyme orientation on the electrode surface and a higher efficiency of the enzymatic network. Also, the decrease in bioelectrode performance prepared at 25 °C can be attributed to the partial loss of enzyme activity [33].

**Figure 2:**
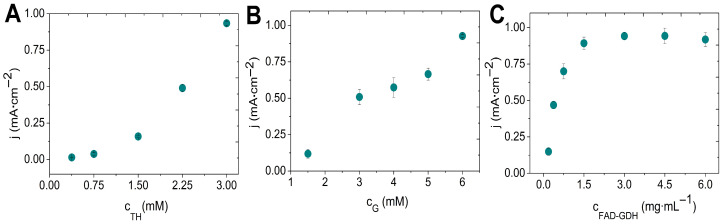
(**A**) Current density vs. TH concentration in crosslinked network determined based on cyclic voltammograms recorded in 200 mM glucose solution in 100 mM PB, pH 8 (*n* = 3, mean ± SD). (**B**) Current density vs. genipin concentration in crosslinking mixture determined based on chronoamperograms recorded in 200 mM glucose solution in 100 mM PB, pH 8, at 0.1 V vs. Ag/AgCl|sat.KCl, 600 s (*n* = 3, mean ± SD). (**C**) Current density vs. FAD-GDH concentration in crosslinking mixture based on chronoamperograms recorded in 200 mM glucose solution in 100 mM PB, pH 8, at 0.1 V vs. Ag/AgCl|sat.KCl, 600 s (*n* = 3, mean ± SD).

Next, the influence of the concentration of thionine, FAD-dependent glucose dehydrogenase, and genipin on bioelectrode performance was examined. As a redox-active compound, thionine mediates the electron transfer between the enzyme active site and the electrode surface. By increasing the concentration of thionine in the crosslinking mixture, the current density generated during glucose oxidation increased (Figure 2A). Then, the influence of genipin concentration was examined. Similar to the mediator, increasing crosslinker concentration improved the performance of the bioelectrode (Figure 2B). Higher genipin concentrations increased crosslink density, improving mediator–enzyme connectivity and current output. Also, a higher concentration of genipin increased the crosslinking degree [39]. Next, the impact of enzyme concentration on bioelectrode performance was studied (Figure 2C). Increasing the concentration of FAD-GDH resulted in generating higher current density during glucose oxidation. The plateau was reached for the prepared bioelectrode, exceeding 3 mg·mL^−1^ FAD-GDH concentration in the final solution. This dependency may result from saturating the crosslinked enzymatic network. However, other limiting factors may also contribute, such as steric hindrance between enzyme molecules at higher concentrations, restricted mass transport of glucose through a densely packed enzymatic layer, or partial enzyme deactivation due to suboptimal orientation or local steric effects. Additionally, increased viscosity of the crosslinking mixture at higher enzyme concentrations could impair uniform deposition and reduce effective contact between the bioactive components and the electrode surface [7,40,41].

### 2.2. Influence of Crosslinker

**Figure 3:**
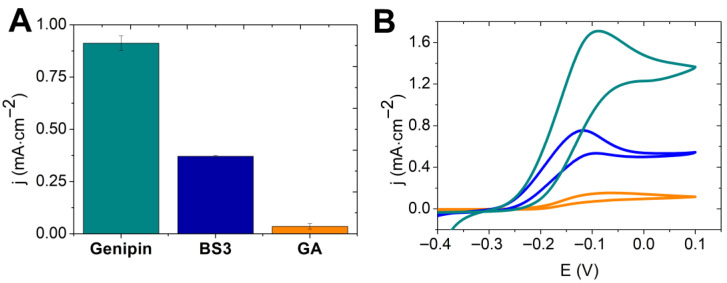
(**A**) Current density generated in 200 mM glucose in 100 mM PB pH 8 by bioelectrodes (*n* = 3, mean ± SD) prepared with different crosslinkers based on chronoamperograms recorded at 0.1 V vs. Ag/AgCl|sat.KCl, 600 s. (**B**) Cyclic voltammograms of bioelectrodes prepared with different crosslinkers recorded in 200 mM glucose in 100 mM PB, pH 8. Scan rate 5 mV·s^−1^.

Three different crosslinking agents were used to prepare the FAD-GDH-thionine network: genipin, BS3, and glutaraldehyde. The influence of a crosslinker on bioelectrode performance was examined using cyclic voltammetry and chronoamperometry in 200 mM glucose in 100 mM PB, pH 8. The catalytic current density generated during glucose oxidation (Figure 3A) by the bioelectrode prepared with genipin was 2.5 and 25 times greater than the current density generated by bioelectrodes prepared with the same concentration of BS3 and GA. Cyclic voltammograms (Figure 3B) recorded for bioelectrodes with genipin and BS3 represent a similar-shaped curve. The spacer length in the BS3 structure is longer compared to other crosslinkers, which can hinder electron transfer. Even though the spacer length of glutaraldehyde is relatively short, the crosslinking reaction rate by GA is much faster than other examined crosslinkers, which can result in blocking of the enzyme active site and explains the different electrochemical response for bioelectrodes prepared with glutaraldehyde.

Additionally, crosslinking by GA may provide an insufficient number of enzyme molecules, as the network formed between thionine and glutaraldehyde is more favorable, thereby limiting the incorporation of enzyme molecules [33]. According to the proposed crosslinking mechanism of genipin [15], its reaction with primary amine groups leads to covalent bond formation involving carbonyl-containing structures. The structures of genipin and thionine, as well as the first two steps of a possible crosslinking mechanism of FAD-GDH and thionine by genipin, are presented in Scheme S2. Unlike networks formed with GA or BS3, genipin-based crosslinked matrices exhibit a more complex chemical architecture and superior biocompatibility. Although the direct role of carbonyl groups in facilitating electron transfer has not been conclusively demonstrated, their presence may support effective integration of redox mediators and favorable enzyme orientation toward the electrode surface. These factors can contribute to enhanced charge transfer efficiency between the enzyme’s active site and the redox mediator, ultimately improving the bioelectrode performance during glucose oxidation [20].

### 2.3. Influence of Electrode Material

**Figure 4:**
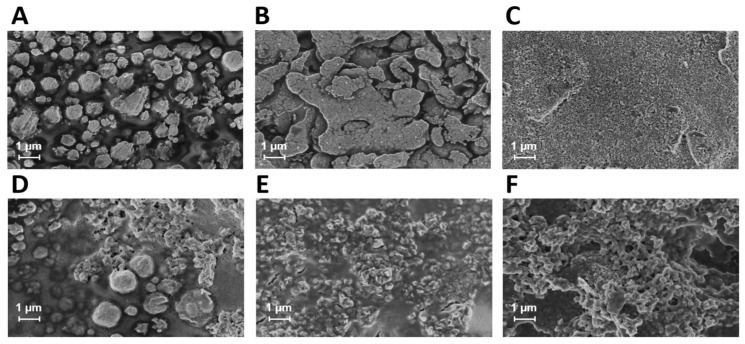
(**A**–**C**) SEM images of gold, silver, and graphite direct-printed electrodes. (**D**–**F**) SEM images of gold, silver, and graphite direct-printed electrodes modified with FAD-GDH-G-TH.

To select an appropriate electrode material for the FAD-GDH-G-TH network, graphite, gold, and silver direct-printed electrodes were examined. The surface characteristics of electrodes before and after modification were examined using scanning electron microscopy (SEM) with energy-dispersive X-ray spectroscopy (EDS). Figure 4 depicts the SEM images of the surface of each electrode before (Figure 4A–C) and after modification by FAD-GDH-G-TH (Figure 4D–F). Images recorded by scanning electron microscope revealed differences in the morphology of electrodes printed with gold, silver, and graphite paste. Most of all, the gold electrode surface (Figure 4A) was inhomogeneous with a bulky roughness (up to 1 µm). Such an uneven surface could be an impeding factor for electron transfer during glucose oxidation and might hinder the uniform biolayer adsorption. The surface of the silver direct-printed electrode (Figure 4B) also had roughness with fractures; however, it was more planar than the gold electrode. The surface of the graphite direct-printed electrode (Figure 4C) appeared to be more uniform, which might result from the smaller grain size of the graphite paste, leading to the formation of the roughness at the sub-micron level and well-developed specific surface area. Such a structure can assure favorable deployment of the biolayer on the surface and shorten the diffusion path, improving the electron transfer during glucose oxidation. SEM images displayed differences on electrode surfaces after modification, confirming biolayer deposition on gold, silver, and graphite direct-printed electrodes.

The differences in the electrodes' composition were reflected by EDS analysis (Figure S3). The unmodified graphite direct-printed electrode (GDPE) contained mainly carbon, with a small content of chlorine (ca. 13.6 wt.%), oxygen (ca. 4.5 wt.%), and sulfur (ca. 0.2 wt.%). The silver direct-printed electrode (AgDPE) before modification contained mainly silver (92.1 wt.%) with small oxygen (4.3 wt.%) and carbon (3.6 wt.%) content, and the unmodified gold direct-printed electrode (AuDPE) contained mainly gold (70.8 wt.%), carbon (21.6 wt.%), and oxygen (7.6 wt.%). Modification of electrodes by FAD-GDH-G-TH increased the levels of oxygen and sulfur on all tested surfaces. The elevated oxygen content was attributable to the presence of genipin and the enzyme, while the sulfur content reflected the inclusion of thionine and the FAD-GDH cofactor. Phosphorus, potassium, sodium, and chlorine were also detected, originating from the phosphate buffer used in the crosslinking mixture. Given the limitations of EDS in detecting low-atomic-number elements, the analysis was considered qualitative for verification of electrodes’ modification with FAD-GDH-G-TH.

**Figure 5:**
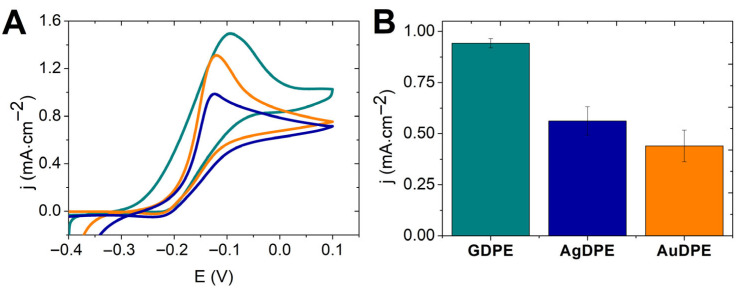
(**A**) Cyclic voltammograms of the FAD-GDH-G-TH on graphite, silver, and gold electrodes in the 200 mM glucose solution. Scan rate 5 mV·s^−1^. (**B**) Current density generated during glucose oxidation on FAD-GDH-G-TH graphite, silver, and gold electrodes (*n* = 3, mean ± SD) based on chronoamperograms recorded at 0.1 V vs. Ag/AgCl|sat.KCl, 600 s.

The electrochemical surface area of the unmodified graphite, gold, and silver direct-printed working electrodes was calculated based on cyclic voltammetry measurements (Figure S4). The electrochemical surface area of gold and graphite electrodes was determined in 10 mM K_3_[Fe(CN)_6_]/K_4_[Fe(CN)_6_] solution in 0.1 M KCl. Due to the silver oxidation occurring at a potential higher than 0.2 V, the electrochemical surface area for the silver direct-printed electrode was determined in the solution of 2 mM [Ru(NH_3_)_6_]Cl_3_ in 0.1 M KNO_3_. Electrochemical surface area for gold, silver, and graphite direct-printed electrodes was calculated by the Randles–Ševčík equation, and they were as follows: 0.033, 0.034, and 0.093 cm^2^, which is consistent with the SEM analysis. Then, the normalization of the catalytic current generated during glucose oxidation was carried out using the electrochemical surface area determined for the particular working electrode. To compare the performance of the FAD-GDH-G-TH bioelectrode on different direct-printed electrodes, the cyclic voltammograms (Figure 5A) and chronoamperograms in 200 mM glucose solution for bioelectrodes prepared on graphite, gold, and silver electrodes were recorded. Current densities (Figure 5B) generated during glucose oxidation clearly showed that the graphite surface was the most suitable for crosslinked FAD-GDH with thionine via genipin. Graphite offered the largest electrochemically active surface area and most favorable biolayer deposition, yielding the highest currents related to the sub-micron-grained structure.

### 2.4. FAD-GDH-G-TH Electrode as Glucose Biosensor

**Figure 6:**
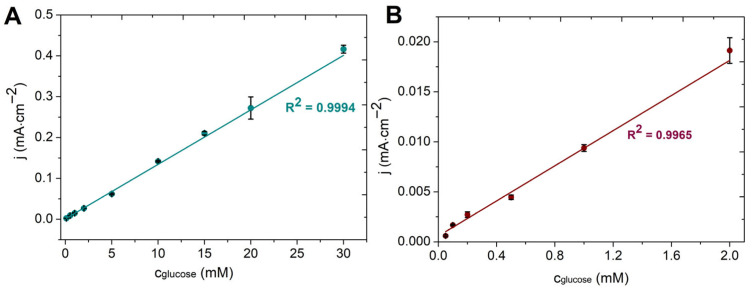
(**A**) Calibration curve for glucose on FAD-GDH-G-TH electrodes (*n* = 3, mean ± SD) in 100 mM PB at pH 8. (**B**) Calibration curve for glucose on FAD-GDH-G-TH electrodes *(n* = 3, mean ± SD) in 100 mM AC at pH 5. The calibration curves were determined based on chronoamperograms recorded at 0.1 V vs. Ag/AgCl|sat.KCl, 600 s.

Electrochemical performance of FAD-GDH-G-TH graphite direct-printed electrode for the glucose determination was evaluated. Electrochemical analysis was performed in a glucose solution prepared in 100 mM AC at pH 5 and 100 mM PB at pH 8, and showed that the prepared bioelectrode has the potential to be used as a glucose biosensor. The linear range between current density and glucose concentration was determined from 0.1 mM to 30 mM at pH 8 (Figure 6A), which covers glucose levels in the majority of body fluids [42]. Prepared bioelectrodes were characterized by a sensitivity of 0.0138 (mA·cm^−2^)·mM^−1^, a 0.043 mM limit of detection (LOD), and a 0.048 mM limit of quantification (LOQ) in PB at pH 8. The LOD and LOQ were determined in the following way: The mean and standard deviation of the blank multiplied by 3 (by 10 for LOQ) were calculated, and then this value was divided by the slope of the calibration curve.

The second calibration curve (Figure 6B) was determined at pH 5 with a narrow glucose concentration range for mimicking the sweat environment required for further biosensor evaluation. The linear range between current density and glucose concentration was determined from 0.05 mM to 2 mM in 100 mM AC at pH 5 (Figure 6B), with a sensitivity of 0.0093 (mA·cm^−2^)·mM^−1^, a 0.013 mM LOD, and a 0.020 mM LOQ. The calibration curves were prepared based on chronoamperometric measurements at 0.1 V vs. Ag/AgCl|sat.KCl (Figure S5).

From an analytical standpoint, the FAD-GDH-G-TH sensor presented in this paper is well within the range needed for both blood and low-glucose biofluids, and its applicability to real samples was evaluated as follows. A series of FAD-GDH-G-TH graphite direct-printed electrodes was fabricated according to the procedure described in Section 3.5. Artificial sweat (AS) solution at pH 5 was prepared based on the recipe outlined in the following publication [43]. The composition of the artificial sweat solution is presented in Table S2. The response of bioelectrodes was measured in an artificial sweat solution by a chronoamperometry technique. The change in current response was proportional to glucose concentration in the tested AS solution. While the actual glucose concentration in the artificial sweat solution was equal to 0.050 mM, the one determined by the FAD-GDH-G-TH electrodes (*n* = 3) was 0.049 ± 0.001 mM. Prepared bioelectrodes demonstrated a high accuracy for glucose detection. The relative errors were determined at the level of 2.12% for bioelectrodes working in an artificial sweat solution at pH 5. Results showed that the prepared bioelectrode has the potential to be implemented for glucose determination in real sample analysis, and interferences like urea, Na^+^, Cl^−^, lactate, alanine, and glycine had no significant impact on the current generated during glucose oxidation on FAD-GDH-G-TH electrodes. The amperometric response of bioelectrodes (*n* = 3) was measured before and after five bending cycles of the electrode to 180° around a 1.6 cm diameter form (Figure S6); bending did not adversely affect bioelectrode performance. Nonetheless, real sweat samples and on-body tests are required for further development and validation.

In the already reported studies, FAD-GDH-based biosensors were designed on unmodified electrodes or modified mainly with carbon nanomaterials [27,44,45,46]. Morshed et al. presented an EBFC-based sensor for glucose detection in the range from 0 to 30 mM and an LOD of 0.28 mM [47]. Another second-generation glucose sensor was prepared by Seo and co-workers based on a synthesized and coordinated polymer with an osmium complex as a mediator. The biosensor exhibited a sensitivity of 0.0015 (mA·cm^−2^)·mM^−1^ and an LOD of 0.003 mM for glucose concentrations between 0.1 and 20.0 mM [26]. However, using osmium complexes and ITO as an electrode limits the practical usage of such sensors in biomedicine. Lee et al. fabricated a biosensor based on direct electron transfer by utilizing a gold-binding peptide (GBP)-fused FAD-GDH from *Burkholderia cepacia.* The biosensor exhibited a linear response in the range from 3 to 30 mM of glucose, with a limit of detection of 3.41 mM [48]. Although the 3–30 mM linear range covers most normoglycemic values and all hyperglycemia, it excludes the clinically relevant hypoglycemic range (<3 mM).

With growing attention to sustainable biosensing materials, replacing toxic aldehyde-based crosslinkers with naturally derived compounds such as genipin aligns with the principles of green analytical chemistry. The LOQ determined for the FAD-GDH-G-TH biosensor in AC pH 5 is 0.02 mM, covering low physiological glucose levels in human sweat [49], making the biosensor a potential candidate for integration in wearable platforms.

### 2.5. Stability of Bioelectrodes

A series of bioelectrodes prepared according to the described procedure in Section 3.5 was used for stability determination. On day “0”, the performance of three bioelectrodes in 200 mM glucose was measured by chronoamperometry at 0.1 V vs. Ag/AgCl|sat.KCl, and the current value was read out in 600 s. The mean value of the signal for the “0” day represents the reference value (100%) for measurements in the next days. The remaining bioelectrodes were stored for 7 days in a refrigerator at 4 °C between measurements. Figure S7 depicts the determined stability of prepared bioelectrodes. From day 0 to day 4, bioelectrodes minimally lost their catalytic activity, resulting in 5% current density decrease, representing good stability during storage. However, from day 5 to 6, an additional 15% loss of current density was observed. The final difference between day 0 and 7 in generated current density during glucose oxidation was ca. 30%.

The performance of bioelectrodes (*n* = 3) stored continuously at 4 °C for 6.5 months after modification with FAD-GDH-G-TH was evaluated. After 6.5 months of storage at 4 °C, the FAD-GDH-G-TH bioelectrodes retained 23.1% (0.209 ± 0.022 mA·cm^−2^) of their initial current density, indicating a gradual loss of performance over time. This decline is likely due to partial enzyme denaturation, increased stiffness and aging of the crosslinked polymer network, and reduced mass and electron transfer efficiency [33]. These findings align with reported long-term behavior of enzymatic bioelectrodes and suggest the need for further optimization of the immobilization matrix to improve stability.

While designing enzyme electrodes, it is essential to consider factors like efficiency and stability. Various approaches have been implemented to improve the network of co-immobilized enzymes with mediators, including entrapment, tethering to a polymer backbone, and crosslinking. Furthermore, the electrode platform significantly affects the performance of enzyme biosensors and EBFCs. Application of nanomaterials increases the electroactive surface area, making numerous active sites available for enzymatic networks [50]. Enzyme stability on the electrode surface can be enhanced by using crosslinkers. A relatively new redox network was developed by crosslinking FAD-GDH with a phenothiazine mediator by PEDGE on GCE [33]. The storage stability of these bioelectrodes was examined at 4 and 25 °C, showing a significant decrease in generated current density after 8 days of storage at room temperature. Meanwhile, bioelectrodes kept at 4 °C show a slight decrease in glucose oxidation current over 8 days, yielding longer stability compared to the redox network obtained in our work. Then, further improvements in the redox network for longer stability will be considered.

## 3. Materials and Methods

### 3.1. Materials

FAD-dependent glucose dehydrogenase (FAD-GDH) from *Aspergillus* sp. (>900 U·mg^−1^ at 37 °C; K_m_ value (Eadie-Hofstee): 5 × 10^−2^ M (D-Glucose) declared by the supplier) was kindly donated by Sekisui Diagnostics (Maidstone, UK). Thionine acetate and bis(sulfosuccinimidyl) suberate (BS3) were purchased from ThermoFisher Scientific (Waltham, MA, USA). To prepare phosphate buffer solutions, potassium phosphate dibasic (≥98%) and dipotassium phosphate monobasic (≥99%) were used. Sodium acetate (≥99%) and acetic acid (≥99%) were used for acetate buffer preparation. Glucose (anhydrous) was used to evaluate the performance of bioelectrodes and as a component of artificial sweat solutions. These analytical-grade reagents, as well as genipin and 25% glutaraldehyde aqueous solution, were purchased from Merck (Darmstadt, Germany). *L*-glycine and sodium chloride from Pol-Aura (Morąg, Poland), *L*-alanine and urea from Merck, and *L*-lactic acid (ThermoFisher Scientific) were used to prepare the artificial sweat solution. Deionized water was obtained by reverse osmosis: 18.2 MΩ (Sartorius (Göttingen, Germany) Arium Comfort). Graphite, gold, silver, and dielectric pastes (C2030519P4, C2041206P2, C2120918P1, and D2070423P5), purchased from SunChemical (Parsippany, NJ, USA), were used for printing electrode systems on polyester foil (MacDermid (Waterbury, CT, USA) Autostat CT7). A red insulating rim surrounding the working electrode was fabricated using 35Z-15R epoxy resin (Solder Chemistry, Landshut, Germany).

### 3.2. Instrumentation and Methods

The electrodes were manufactured by the 3-Axis PROPlus Series Automated Fluid Dispensing Robot from Nordson (Westlake, OH, USA). For the electrochemical study, a potentiostat/galvanostat PalmSens system (Houten, The Netherlands) was used. Thionine acetate and bioelectrode characteristics were studied by cyclic voltammetry (CV) and chronoamperometry (CA). All electrochemical measurements with graphite, gold, and silver direct-printed working electrodes were performed vs. Ag/AgCl|sat.KCl reference electrode (Redoxme AB, Norrköping, Sweden) and platinum auxiliary electrode (ItalSens from PalmSens BV Houten, The Netherlands). Surface analysis of electrodes before and after modification was carried out using a scanning electron microscope ZEISS (Oberkochen, Germany) CrossBeam 540, equipped with an energy-dispersive spectroscopic (EDS) detector (Bruker, Billerica, USA).

### 3.3. Manufacture of Direct-Printed Electrodes

The electrodes were fabricated on a polyester foil with a thickness of 175 µm (MacDermid Autostat CT7). A conductive track was first printed using silver paste, followed by deposition of a polymeric insulating layer. Subsequently, the working electrode area—composed of graphite, gold, or silver paste—was printed in a defined, rounded geometry on top of the silver path. Each printed layer was thermally cured in a laboratory oven at 130 °C for 3 h prior to the deposition of the next layer. The epoxy resin rim around the working electrode was printed to prevent the biolayer from flowing out from the electrode and to ensure a consistent geometrical surface area and even coverage by the enzymatic network.

### 3.4. Electrochemical Characteristic of Direct-Printed Electrodes

The electrochemical characterization of unmodified graphite and gold direct-printed electrodes was performed by cyclic voltammetry in a 10 mM K_3_[Fe(CN)_6_]/K_4_[Fe(CN)_6_] solution in 0.1 M KCl. Characterization of unmodified silver direct-printed electrodes was performed in 2 mM [Ru(NH_3_)_6_]Cl_3_ in 0.1 M KNO_3_, as silver oxidation occurs at potentials above 0.2 V. Cyclic voltammograms were recorded for each type of electrode (*n* = 3) at a scan rate of 100 mV·s^−1^. The electrochemical surface area of electrodes was calculated using the Randles–Ševčík equation, based on the peak currents obtained from cyclic voltammetry and assuming diffusion coefficients 6.4 × 10^−6^ cm^2^·s^−1^ [51] and 8.0 × 10^−6^ cm^2^·s^−1^ [52] for the ferrocyanide and ruthenium redox probes, respectively.

### 3.5. Bioelectrode Preparation

To increase the solubility of thionine acetate (TH), a 40 mM TH solution was prepared in 100 mM AC at pH 5, incubated, and rotated in an orbital shaker at 50 °C for one hour. Then, the solution was diluted 10 times with 100 mM PB at pH 7, obtaining a 4 mM solution of thionine acetate.

An enzymatic network was prepared by dissolving 4 mg·mL^−1^ FAD-GDH from *Aspergillus* sp. (declared activity > 900 U·mg^−1^) in the above-described 4 mM thionine acetate solution and mixed at a 3:1 *v*/*v* ratio with 24 mM crosslinker, genipin, BS3, or glutaraldehyde, prepared in 100 mM PB at pH 7. It is important to note that the initial genipin (G) solution was oversaturated; however, complete dissolution was achieved after a three-fold dilution. The final concentrations of each component after dilution were as follows: 3 mg·mL^−1^ FAD-GDH, 3 mM thionine, and 6 mM crosslinker. Then, 8 µL of the prepared solution was drop-casted on the direct-printed electrode surface and left to dry at 4 °C for 16 h. Before each measurement, bioelectrodes were rinsed in buffer solution to remove unbound molecules from the electrode surface.

### 3.6. Biosensor Characterization

A series of graphite direct-printed bioelectrodes was fabricated according to the procedure described above. The glucose solutions in the range from 0.05 to 2 mM and from 0.1 to 30 mM were prepared in 100 mM AC at pH 5 and in 100 mM PB at pH 8, respectively. The catalytic current generated by bioelectrodes (*n* = 3) in solutions with different glucose concentrations and in buffer solutions (blank) was determined by chronoamperometry measurements at 0.1 V vs. Ag/AgCl|sat.KCl with a readout value at 600 s. The catalytic current density generated during glucose oxidation was calculated by dividing the current by the electrochemical surface area of direct-printed electrodes.

### 3.7. Determination of Glucose in Artificial Sweat Solution

An artificial sweat solution containing 0.05 mM of glucose at pH 5 was prepared. The specific composition of the artificial sweat solution is detailed in Table S2. A series of graphite direct-printed bioelectrodes was fabricated according to the procedure described in Section 3.5. Then, chronoamperograms for bioelectrodes (*n* ≥ 3) were recorded in artificial sweat solutions at 0.1 V vs. Ag/AgCl|sat.KCl with the readout current value set for 600 s.

### 3.8. Determination of the Storage Stability

A series of 21 graphite direct-printed electrodes (3 electrodes per day) were modified by the FAD-GDH-G-TH mixture in the same manner as described in Section 3.5. The next day, set as day “0”, the performance of three random electrodes was determined by chronoamperometry at 0.1 V vs. Ag/AgCl|sat.KCl in a 200 mM glucose solution in 100 mM PB, pH 8, and the current value was read out in 600 s. The remaining bioelectrodes were stored in a refrigerator at 4 °C before measurements on a particular day. The mean value of the signal for day “0” represents the reference value (100%) for measurements in the next days.

## 4. Conclusions

In this study, a high-performance, flexible bioelectrode was developed by direct printing and one-step enzymatic crosslinking using genipin. The FAD-GDH-G-TH bioelectrode achieved a catalytic current density of 0.942 mA·cm^−2^ during glucose oxidation. To the best of our knowledge, so far, the highest current density of 0.830 mA·cm^−2^ generated by FAD-GDH and crosslinked with thionine via NHS-based agent on a bare electrode was reported by Hossain et al. [36]. The use of genipin as a biocompatible crosslinker significantly enhanced electron transfer efficiency, compared to bioelectrodes prepared with glutaraldehyde and BS3. The resulting bioelectrode exhibited a linear response to glucose in the physiologically relevant range of 0.048–30 mM at pH 8 and 0.02–2 mM at pH 5 and demonstrated an accurate detection range in artificial sweat solution. Therefore, the developed bioelectrode can be integrated with the biocathode or abiotic cathode into power generation systems or into electrochemical glucose-sensing platforms. Direct printing on flexible polyester substrate and using biocompatible crosslinkers highlights the potential for bioelectrode integration into wearable electrochemical systems. Nevertheless, further improvements in long-term stability will be required to support real-world deployment.

## Data Availability

The data presented in this study are available on request from the corresponding author.

## Supplementary Materials

The following supporting information can be downloaded at https://www.mdpi.com/article/10.3390/molecules31020327/s1: Figure S1: Midpoint potential of free thionine vs. pH of buffer solution and midpoint potential of thionine crosslinked in FAD-GDH-G-TH vs. pH of buffer solution; Figure S2: (A) Current density vs. pH of crosslinking mixture determined based on cyclic voltammograms recorded in 200 mM glucose in PB pH 8. (B) Current density vs. pH of glucose solution determined based on chronoamperograms recorded at 0.1 V vs. Ag|AgCl (KCl sat.). The current value was readout at 600 s; Figure S3: EDS spectra recorded for gold, silver and graphite direct printed electrodes before (A–C) and after modification of FAD-GDH-G-TH (D–F); Figure S4: Cyclic voltammograms recorded in 10 mM K_3_[Fe(CN)_6_]/K_4_[Fe(CN)_6_] solution in 0.1 M KCl for graphite (GDPE) and gold (AuDPE) direct printed electrodes and in 2 mM [Ru(NH_3_)_6_]Cl_3_ in 0.1 M KNO_3_ for the silver (AgDPE) direct printed electrode. Scan rate 0.1 V·s^−1^; Figure S5: Chronoamperograms recorded at 0.1 V vs. Ag|AgCl (KCl sat.) in glucose solution in 100 mM AC pH 5 (A) and 100 mM PB pH 8 (B) based on the calibration curves were prepared. The current value was readout at 600 s; Figure S6: Exemplary chronoamperogram recorded at 0.1 V vs. Ag|AgCl (KCl sat.) in 20 mM glucose solution in 100 mM PB pH 8 before and after 5 bending cycles; Figure S7: Long term storage stability of FAD-GDH-G-TH bioelectrodes. Measurements performed in 200 mM glucose in 100 mM PB pH 8; Table S1: Comparison of current density generated during glucose oxidation by different FAD-GDH-based bioelectrodes; Table S2: Composition of artificial sweat solution; Scheme S1: Schematic illustration of the electron transfer pathway during glucose oxidation on the electrode surface; Scheme S2: Schematic illustration of the possible mechanism of crosslinking FAD-GDH and thionine by genipin in neutral condition [53,54,55,56].
