# Supplementary material for: Genipin as an Effective Crosslinker for High-Performance and Flexible Direct-Printed Bioelectrodes
Source: Molecules. 2026 Jan 17;31(2):327. doi: 10.3390/molecules31020327 (PMC12843758; doi:10.3390/molecules31020327)
Supplement: Supplementary file 1 [file molecules-31-00327-s001.zip › molecules-4021874-supplementary.pdf]

Supplementary data

Genipin as an Effective Crosslinker for High-Performance and Flexible Direct-Printed Bioelectrodes

*Kornelia Bobrowska *, Marcin Urbanowicz , Agnieszka Paziewska-Nowak , Marek Dawgul , Kamila Sadowska*

Nalecz Institute of Biocybernetics and Biomedical Engineering, Polish Academy of Sciences, Ks. Trojdena 4 St., 02-109 Warsaw, Poland

*Corresponding author: kbobrowska@ibib.waw.pl

Table S1. Comparison of current density generated during glucose oxidation by different FAD-GDH-based bioelectrodes.

**Electrode**	**Mediator**	**Immobilization component**	**Current density ($mA \cdot cm^{-2}$)**	**$C_{glucose}$ (mM)**	**Ref.**
GC-MWCNTs	1,2-NQ-4-glycidyl	linear PEI	1.95	100	[1]
GC	$[Ru(PQ)_2(NH_2$-phen)$](NH_3)_2$	PDDA	0.036	100	[2]
GC	2-carboxyethyl-3-nitro-1,4-NQ	branched PEI	1.97	50	[3]
Graphite disc-MWCNTs	$[P20$-$Os(dmobpy)_2$ (4-AMP)Cl$].PF_6$	PEDGE	0.22	5	[4]
GC-MWCNTs	Azure-A	Adsorption	5.6	100	[5]
Buckypaper	Thionine	polynorbornene-pyrene-NHS polymer	3.7	50	[6]
SPC	Thionine	PEDGE	0.26	40	[7]
GC	Thionine	PEDGE	0.40	200	[8]
GC	Thionine	NHS-based crosslinker	0.83	200	[9]
GDP	Thionine	Genipin	0.94	200	This work

Table S2. Composition of artificial sweat solution [10].

**Artificial sweat solution pH 5**	
**Component**	**Concentration (mM)**
Urea	10
Sodium chloride	31
Lactic acid	14
Glucose*	0.05
Alanine	0.36
Glycine	0.39

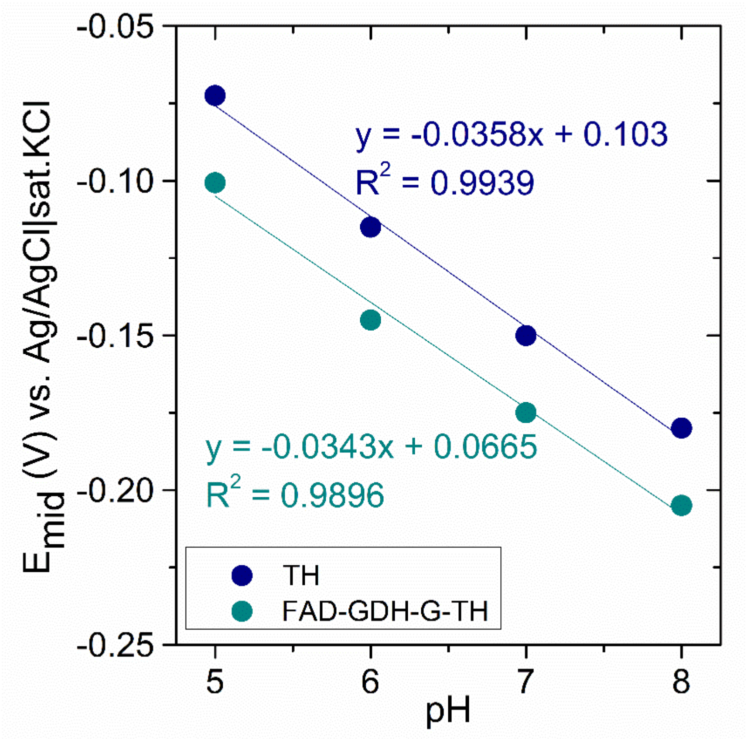

Figure S1. Midpoint potential of free thionine vs. pH of buffer solution and midpoint potential of thionine crosslinked in FAD-GDH-G-TH vs. pH of buffer solution.

Scheme S1. Schematic illustration of the electron transfer pathway during glucose oxidation on the electrode surface.

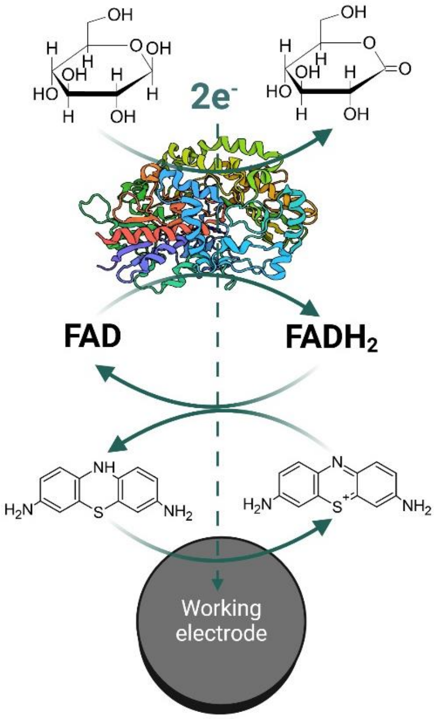

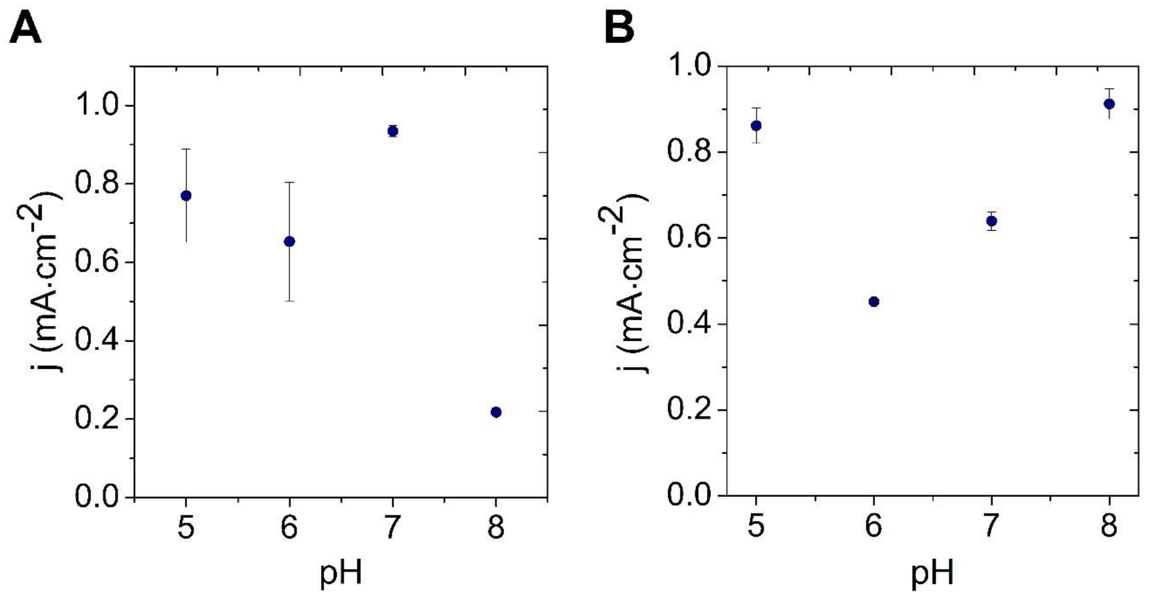

Figure S2. (A) Current density vs. pH of crosslinking mixture determined based on cyclic voltammograms recorded in 200 mM glucose in PB pH 8. (B) Current density vs. pH of glucose solution determined based on chronoamperograms recorded at 0.1 V vs. Ag|AgCl (KCl sat.). The current value was readout at 600 s.

Scheme S2. Schematic illustration of the possible mechanism of crosslinking FAD-GDH and thionine by genipin in neutral condition [11].

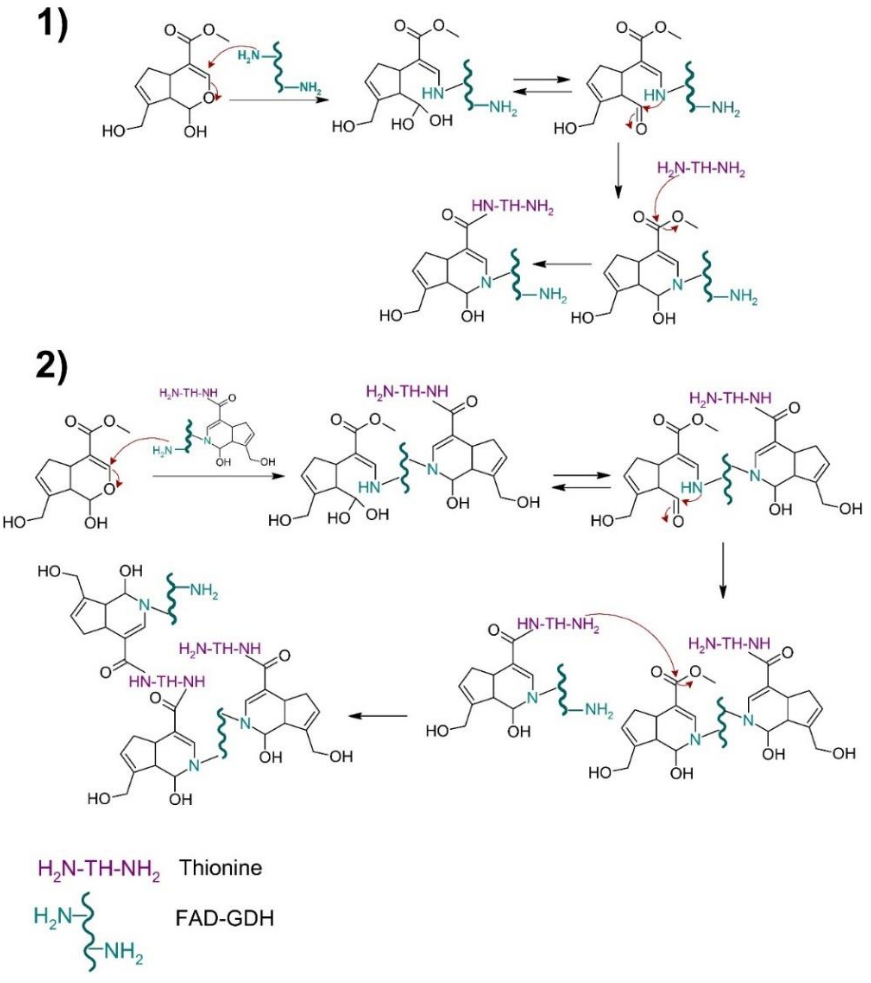

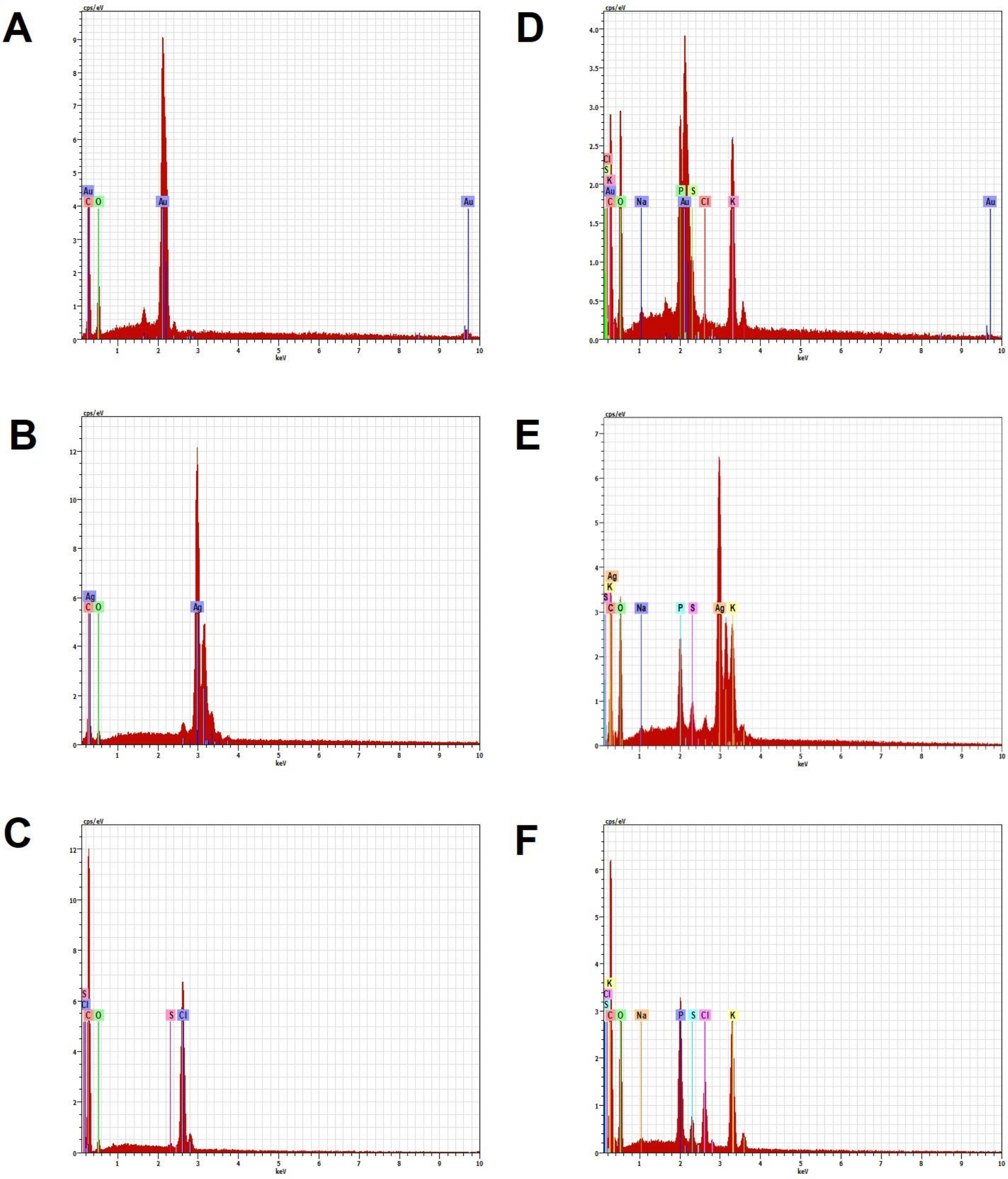

Figure S3. EDS spectra recorded for gold, silver and graphite direct printed electrodes before (A-C) and after modification of FAD-GDH-G-TH (D-F).

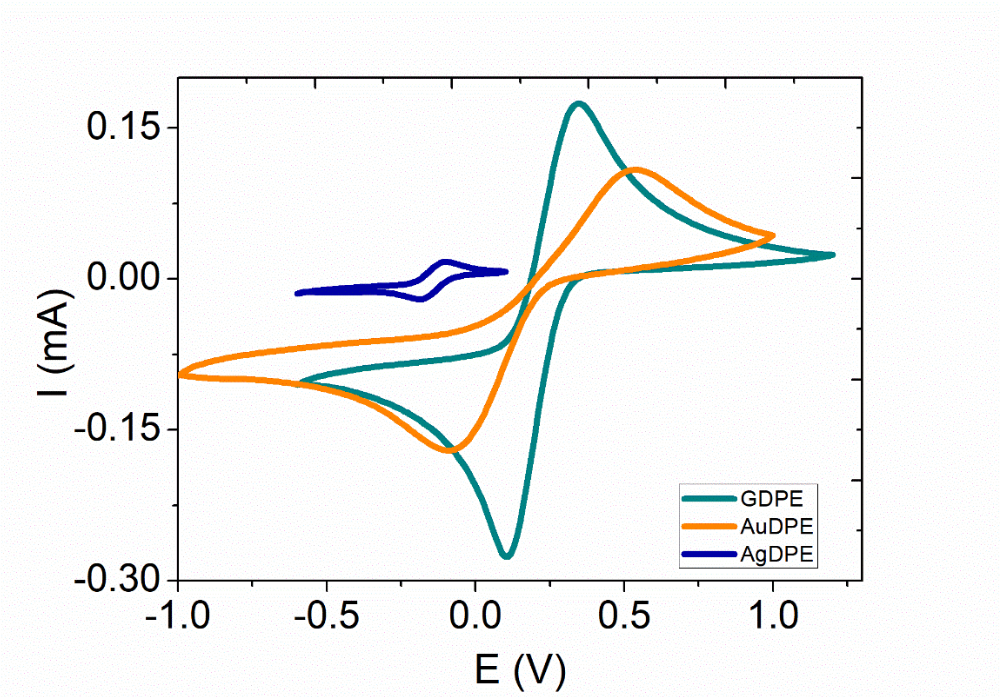

Figure S4. Cyclic voltammograms recorded in 10 mM $K_3[Fe(CN)_6]/K_4[Fe(CN)_6]$ solution in 0.1 M KCl for graphite (GDPE) and gold (AuDPE) direct printed electrodes and in 2 mM $[Ru(NH_3)_6]Cl_3$ in 0.1 M KNO_3 for the silver (AgDPE) direct printed electrode. Scan rate 0.1 $V{\cdot}s^{-1}$.

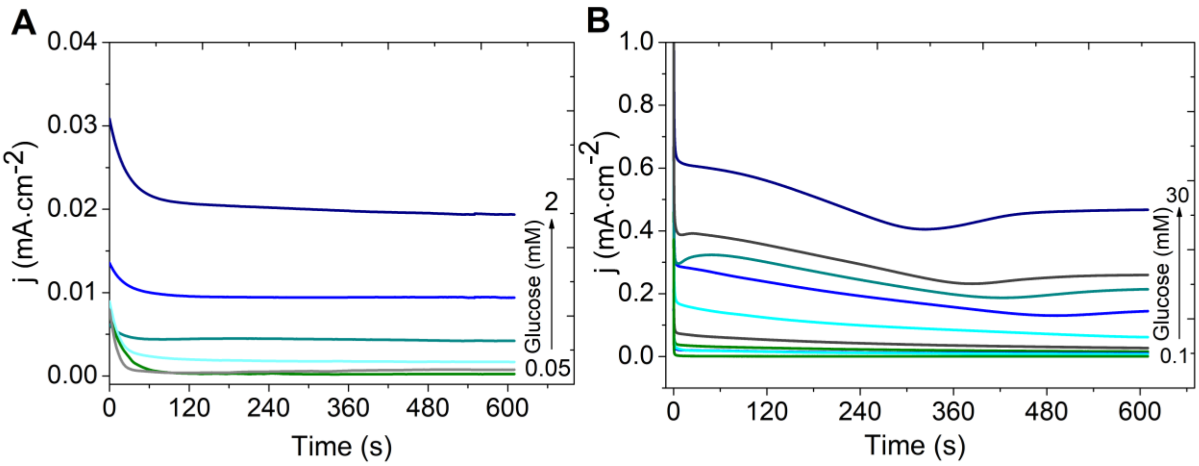

Figure S5. Chronoamperograms recorded at 0.1 V vs. Ag|AgCl (KCl sat.) in glucose solution in 100 mM AC pH 5 (A) and 100 mM PB pH 8 (B) based on the calibration curves were prepared. The current value was readout at 600 s.

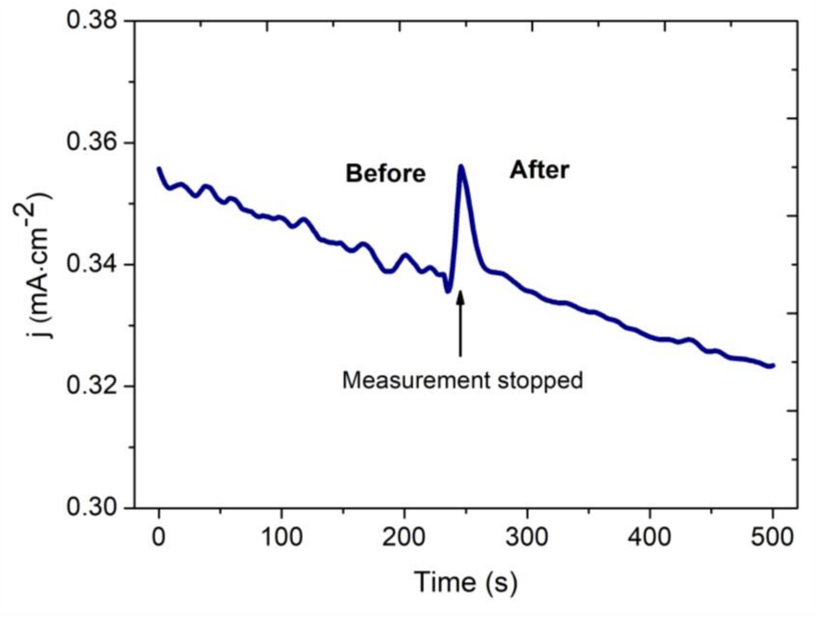

Figure S6. Exemplary chronoamperogram recorded at 0.1 V vs. Ag|AgCl (KCl sat.) in 20 mM glucose solution in 100 mM PB pH 8 before and after 5 bending cycles.

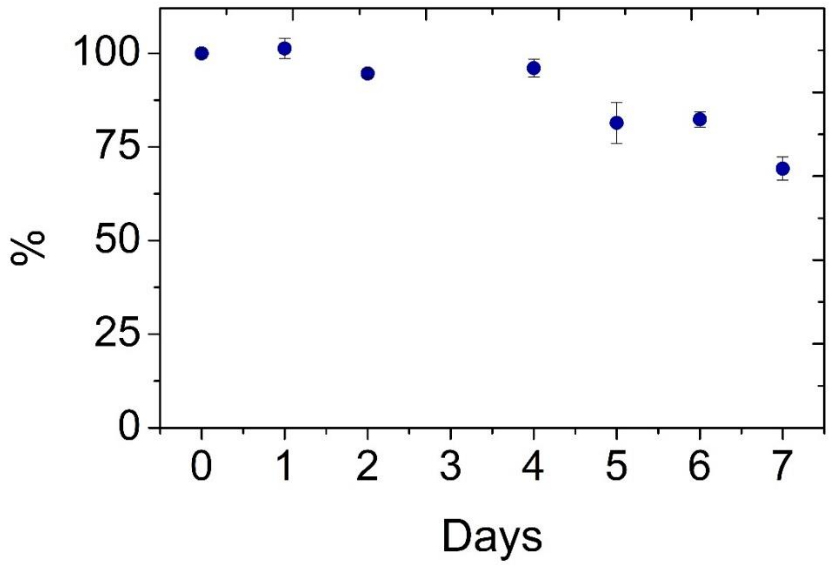

Figure S7. Long term storage stability of FAD-GDH-G-TH bioelectrodes. Measurements performed in 200 mM glucose in 100 mM PB pH 8.

Literature

1. Milton, R.D.; Hickey, D.P.; Abdellaoui, S.; Lim, K.; Wu, F.; Tan, B.; Minteer, S.D. Rational Design of Quinones for High Power Density Biofuel Cells. *Chem. Sci.* **2015**, *6*, 4867–4875, doi:10.1039/C5SC01538C.

2. Sakuta, R.; Takeda, K.; Ishida, T.; Igarashi, K.; Samejima, M.; Nakamura, N.; Ohno, H. Multi-Enzyme Anode Composed of FAD-Dependent and NAD-Dependent Enzymes with a Single Ruthenium Polymer Mediator for Biofuel Cells. *Electrochem. commun.* **2015**, *56*, 75–78, doi:10.1016/j.elecom.2015.04.013.

3. Hou, C.; Lang, Q.; Liu, A. Tailoring 1,4-Naphthoquinone with Electron-Withdrawing Group: Toward Developing Redox Polymer and FAD-GDH Based Hydrogel Bioanode for Efficient Electrocatalytic Glucose Oxidation.

Electrochim. Acta **2016**, *211*, 663–670, doi:10.1016/j.electacta.2016.06.078.

4. Ó Conghaile, P.; Pöller, S.; MacAodha, D.; Schuhmann, W.; Leech, D. Coupling Osmium Complexes to Epoxy-Functionalised Polymers to Provide Mediated Enzyme Electrodes for Glucose Oxidation. *Biosens. Bioelectron.* **2013**, *43*, 30–37, doi:10.1016/j.bios.2012.11.036.

5. Tsujimura, S.; Tanaka, S.; Gross, A.; Holzinger, M. Electrochemical Modification at Multiwalled Carbon Nanotube Electrodes with Azure A for FAD- Glucose Dehydrogenase Wiring: Structural Optimization to Enhance Catalytic Activity and Stability. *J. Phys. Energy* **2021**, *3*, 024004, doi:10.1088/2515-7655/abd298.

6. Fritea, L.; Gross, A.J.; Gorgy, K.; O'Reilly, R.K.; Le Goff, A.; Cosnier, S. A Bifunctional Triblock Polynorbornene/Carbon Nanotube Buckypaper Bioelectrode for Low-Potential/High-Current Thionine-Mediated Glucose Oxidation by FAD-GDH. *J. Mater. Chem. A* **2019**, *7*, 1447–1450, doi:10.1039/C8TA10644D.

7. Morshed, J.; Hossain, M.M.; Zebda, A.; Tsujimura, S. A Disposable Enzymatic Biofuel Cell for Glucose Sensing via Short-Circuit Current. *Biosens. Bioelectron.* **2023**, *230*, 115272, doi:10.1016/j.bios.2023.115272.

8. Hossain, M.M.; Morshed, J.; Tsujimura, S. Designing a Cross-Linked Redox Network for a Mediated Enzyme-Based Electrode. *Chem. Commun.* **2021**, *57*, 6999–7002, doi:10.1039/D1CC01707A.

9. Hossain, M.M.; Rezki, M.; Shalayel, I.; Zebda, A.; Tsujimura, S. Effects of Cross-Linker Chemistry on Bioelectrocatalytic Reactions in a Redox Cross-Linked Network of Glucose Dehydrogenase and Thionine. *ACS Appl. Mater. Interfaces* **2024**, *16*, 44004–44017, doi:10.1021/acsami.4c08782.

10. Watson, F.; Keevil, C.W.; Chewins, J.; Wilks, S.A. Artificial Human Sweat as a Novel Growth Condition for Clinically Relevant Pathogens on Hospital Surfaces. *Microbiol. Spectr.* **2022**, *10*, doi:10.1128/spectrum.02137-21.

11. Shalayel, I.; Vallee, Y.; Zebda, A. Study of Genipin Behavior in Neutral and Acidic Aqueous Solutions at Elevated Temperatures. *ChemistrySelect* **2023**, *8*, doi:10.1002/slct.202204705.
